# Donor-Recipient Size Mismatch in Paediatric Renal Transplantation

**DOI:** 10.1155/2014/317574

**Published:** 2014-02-13

**Authors:** J. Donati-Bourne, H. W. Roberts, R. A. Coleman

**Affiliations:** ^1^Department of Urology, Birmingham Children's Hospital, The Birmingham Children's Hospital NHS Foundation Trust, Steelhouse Lane, Birmingham B4 6NH, UK; ^2^Department of Ophthalmology, Peterborough City Hospital, Peterborough and Stamford Hospitals NHS Foundation Trust, Peterborough PE3 9GZ, UK

## Abstract

*Introduction*. End stage renal failure in children is a rare but devastating condition, and kidney transplantation remains the only permanent treatment option. The aim of this review was to elucidate the broad surgical issues surrounding the mismatch in size of adult kidney donors to their paediatric recipients. *Methods*. A comprehensive literature search was undertaken on PubMed, MEDLINE, and Google Scholar for all relevant scientific articles published to date in English language. Manual search of the bibliographies was also performed to supplement the original search. *Results*. Size-matching kidneys for transplantation into children is not feasible due to limited organ availability from paediatric donors, resulting in prolonged waiting list times. Transplanting a comparatively large adult kidney into a child may lead to potential challenges related to the surgical incision and approach, vessel anastomoses, wound closure, postoperative cardiovascular stability, and age-correlated maturation of the graft. *Conclusion*. The transplantation of an adult kidney into a size mismatched paediatric recipient significantly reduces waiting times for surgery; however, it presents further challenges in terms of both the surgical procedure and the post-operative management of the patient's physiological parameters.

## 1. Introduction

Paediatric end stage renal failure (ESRF) remains a relatively rare condition in children, with an estimated incidence of 11-12 per million per year and prevalence of 60 per million age-related population in Europe [1]. Epidemiological statistics have been stable over the last decade in developed countries; however, evidence suggests that it remains an underdiagnosed condition in third-world countries [1].

ESRF has very serious implications for the patient with significant morbidity and increased mortality. Multiple medical comorbidities often coexist with paediatric renal failure and the patient and his or her family also face multiple hospital attendances and admissions, which may be further complicated by the patients' limited understanding of their own condition, particularly if very young [2].

Treatment options available include either renal replacement therapy or kidney transplantation. Renal replacement therapy can be achieved via haemodialysis, which requires multiple weekly attendances via a long term central line or the surgical creation of an arteriovenous fistula, both being potential conduits for infection. The alternative is peritoneal dialysis, which is more commonly used in children compared to adults, as it allows for dialysis to be performed at home [3].

Dialysis is associated with a significantly worse quality of life when compared to transplantation, and it confers a fourfold increase in mortality risk to the child [4]. Transplantation is therefore the preferable option in most cases. While transplantation is the preferred long term treatment modality, the lay public often overlooks the associated significant short and long term risks, ranging from those immediately related to surgery and anaesthesia to the complications of rejection and wide-reaching side-effects of immunosuppressant therapy [5].

When applied to children, there are further complications to consider in renal transplantation, ranging from the limited number of potential age-matched donors in developed countries to the relative lack of space in the young paediatric abdominal cavity to accommodate an extra solid organ [6].

This review was undertaken to explore the issues surrounding the size mismatch between adult and paediatric kidneys, with the aim of illustrating strategies for managing this increasing problem in the field of paediatric surgery. To our knowledge, no similar such systematic review has been published.

## 2. Methods

A comprehensive literature search was undertaken on MEDLINE, PubMed, and Google Scholar for all scientific articles published in the English language. No time frame for publication was set. Keywords for search included paediatric, renal failure, kidney transplantation, size, adult donor, and mismatch. These were also used in combination with the Boolean operators: AND and OR. The literature search was further extended by applying the “related article” function. The bibliography sections of the generated articles were further searched to identify articles which had been missed by the initial electronic search.

## 3. Discussion

### 3.1. Limitations in Organ Availability

One of the most distressing elements for transplant candidates is the time spent on the transplant waiting list, and it is not uncommon that this time of uncertainty may last for many years. The media have attempted to raise awareness of this problem by promoting the Organ Donation Card system and other similar campaigns, and the British government has at length discussed the proposal of adopting an “opt-out” system to increase the availability of organs for transplantation; nonetheless, there still currently remains an organ shortage to meet the increasing demands of our growing population.

Paediatric patients only occupy 1% of all patients on the Eurotransplant waiting list for a kidney [7]; however, in light of the current scarcity of available organ donors, further ultraselection of kidney donors for children such that these are age- and size matched was shown to lead to disproportionately long waiting times for very young recipients [8].

It is further surprising that even when a kidney from a paediatric donor is retrieved, it will not be primarily allocated to a paediatric recipient on the Eurotransplant system [9].

In the USA, however, the United Network for Organ Sharing (UNOS) initiated an allocation policy in 2005 called “Share 35” which preferentially allocated kidneys from deceased donors aged <35 years to paediatric recipients aged <18 years. The aim was to reduce paediatric waiting times and increase high-quality donors for them [10].

Nonetheless, the use of adult sized kidneys may provide a valid alternative to tackle the shortfall of donors, particularly as many children on the renal transplant list will have one or both parents willing to act as a living related donor. This solution, however, poses the surgical team with a far more technically challenging task than the conventional size matched transplantation procedure, whilst also posing further increased risks of graft malfunction which jeopardises the overall outcome for the patient [7].

### 3.2. Cardiovascular Complications of Size Mismatch

A donated adult kidney is adapted to the adult cardiovascular system, yet following transplantation must resume function in the recipient child's cardiovascular system. The two systems have many fundamental differences and these can cause significant disruptions.

The kidneys are important regulators of blood pressure and at rest receive one fifth (20%) of an adult's cardiac output. For a 70 kg adult male with an estimated blood volume of 5 litres, at rest the kidneys will be perfused with approximately 500 mL of blood per minute each (10% + 10%). If one kidney was hypothetically transplanted into a small child with a body mass of 10 kg and estimated total blood volume of 1 litre, in order to maintain that same level of perfusion it had in the adult donor, half the child's cardiac output would have to be directed to that transplanted kidney, a scenario which is clearly not sustainable. This commonly results in graft hypoperfusion and nonfunction, further complicated by the significantly lower resting blood pressure maintained in small children compared to the adult in which the donated kidney was formerly adapted to. This redirection of blood flow and dangerous resulting hypotension is difficult to manage and may require large amounts of intravenous fluids as well as the concomitant use of inotropes [7].

Studies that focused on the cardiovascular benefits that transplantation provides have shown that transplantation early in ESRF reduces the known cardiovascular risk factors associated with chronic kidney disease and long term dialysis [11]. Cardiovascular disease is responsible for the deaths of 36% of all children with end stage renal failure, 34% of those on dialysis and 11% of paediatric deaths after transplantation [12].

### 3.3. Graft Growth and Senescence

Dubourg et al. [13] demonstrated that an already mature adult graft will initially adapt to the paediatric recipient following transplantation; however, thereafter, it does not increase its filtration function to correlate with the increasing size and filtration demand of the growing child. Conversely, the absolute glomerular filtration rate (GFR) of a kidney from a paediatric donor will increase to match the child recipient's body growth, leading to a stable GFR in the recipient for many years after transplantation.

The authors reached this conclusion based on their study which followed up a cohort of 134 children who had received a kidney from either an adult or a child, and whose absolute and relative GFRs were calculated at yearly intervals for up to eight years. A statistically significant difference between the two groups was found from beyond the six-month posttransplant mark, whereby the relative GFRs in the children receiving paediatric grafts were greater than those receiving an adult graft, the latter group indeed showing a gradual decline in GFR. This difference became greater with the increasing number of years after transplant.

The key question is whether this difference is simply due to the donated kidney's mass or rather to the number of functioning nephrons in the graft, which could be interpreted as a “senescence” of a kidney, whereby the increasing age of the donor may result in there being proportionally less functioning nephrons as well as less of a response to endogenous growth factors [14].

### 3.4. Choice of Surgical Incision

The choice of which incision to perform will pose the first potential surgical issue. An infant or small child has a comparatively small abdomen and thus operative field, and for a transplant to be safely undertaken with reasonable access, a midline laparotomy incision may be necessary, as opposed to the standard transverse oblique Gibson incision used in adults, which skirts the inguinal ligament superiorly.

Midline laparotomy provides quick access and reduces operative time as well as providing excellent view for this particularly challenging procedure. However, it may lead to more post-operative complications, such as higher rates of incisional hernias as well as post-operative pain when compared to transverse or oblique incisions [15]. Filocamo et al. in turn proposed that the midline incision poses a lower risk of incisional hernias when compared to the J-shaped incision, another technique sometimes used for renal transplantation [16].

### 3.5. Surgical Approach

The traditional approach used in small children receiving an adult donor kidney was for the organ to be placed within the peritoneal cavity, which gave the advantage of improved access to the great vessels as well as an increased working space for the surgeon to perform safe vascular anastomoses [17].

More recently the extraperitoneal approach has increased in popularity, and one study suggested that this new method has several advantages over the traditional procedure, including preservation of the peritoneal cavity for future dialysis if this were to become necessary again, as well as minimising the risks of future adhesional bowel obstruction in the recipient. Furthermore, any collections such as post-operative abscesses, urinomas, and chyle leaks would be self-contained in the extraperitoneal space and therefore more likely to be amenable to percutaneous drainage [18]. Other studies in the literature which directly compare the intraperitoneal and extraperitoneal approaches have not revealed any significant differences in the overall rates of surgical complications [19, 20].

Heap et al. conducted a retrospective study from 2011 to establish whether there is a difference in renal function and outcome of the transplant based on the selected intra- versus extraperitoneal approach. Thirty children under the age of six years who had received a transplant at the unit in Manchester, UK, were retrospectively reviewed with the studied outcome being graft survival and function. This series revealed an early improved function in the patients who had undergone an extraperitoneal approach, however no long term differences were found between the two groups [18].

### 3.6. Size Mismatch in Vascular Anastomosis

In adults, the donor's renal artery is most frequently anastomosed either to the recipient's external iliac artery in an end-to-side fashion, or to the recipient's internal iliac artery in an end-to-end fashion [21]. If the kidney is from a cadaveric donor, a Carrel aortic patch may be concomitantly taken to facilitate the anastomosis procedure.

For larger children, the anastomosis procedure does not differ significantly from the adult technique [22]. In infants and young children, however, this may not be possible, as these patients may not yet be weight-bearing with the resultant increase in size of vessels supplying the lower limb, making these unsuitable for anastomosis. The alternative strategy is to use a larger vessel such as the aorta and the common iliac artery and the inferior venae cava for venous anastomosis [23].

Rickard et al. [24] recently investigated and contrasted the techniques for managing a small to large diameter discrepancy in an arterial anastomosis, using animal models. Two techniques were directly compared for patency and revision rates. The first technique involved obliquely sectioning the smaller vessel to produce one with a greater circumference, and the second technique involved invaginating the smaller vessel inside the larger one. The authors found no statistically significant difference in patency rates between the two strategies; however, the oblique sectioning technique took longer to perform and was associated with a greater risk of requiring subsequent revision.

### 3.7. Abdominal Wall Closure

In donor-recipient mismatch, significant problems with wound closure may arise. When placed in a small paediatric abdomen, an adult kidney may be so comparatively large as to make primary wound apposition and abdominal closure impossible to achieve, made even more difficult by oedema of the abdominal contents. This excessive pressure can potentially lead to abdominal compartment syndrome, jeopardising graft perfusion [25].

There are several methods available to overcome issues with abdominal wall closure. The wound may be left open, but this is associated with a poorer outcome [26], or alternatively the gap may be bridged by using a muscle flap or a mesh [27]. Mesh materials include those made of extracellular matrix or natural porcine derivatives, and the choice of which type to use is debatable, and indeed practice may vary between departments. Studies have compared the different types of mesh material, and porcine-derived implants have been shown to have excellent success rates [25, 28].

### 3.8. Outcome-Graft Survival

The ultimate question in paediatric renal transplantation is whether paediatric recipients fare better with adult or paediatric donor grafts. Since the 1980s, there have been studies directly comparing graft survival and outcome in children receiving kidneys from adult versus paediatric donors, and the conflicting findings from these have caused difficulty in reaching a consensus amongst nephrologists. Earlier reports showed shorter graft survival for kidneys from paediatric donors due to increased infection and complication rate [29–31].

Hayes et al. [31] retrospectively studied 126 paediatric cadaveric kidneys transplanted over a 10-year period, comparing one-year patient and graft survival for their paediatric groups against a selected adult-to-adult donor control group. Results showed a comparable functional outcome with the control, but a higher incidence of infections and complications in the young donor age groups.

As a result of early reports from the 1980s and 90s, paediatric donor organs were often not accepted for paediatric recipients because they had been associated with inferior outcome in graft function. However, subsequent reports [32, 33] suggested that kidneys from adult donors transplanted into children downregulate filtration and may not increase their function to correlate with body growth over the years.

Pape et al. [9] compared long term graft survival and kidney function of adult versus paediatric grafts transplanted into ninety-nine children under the age of 10 years. The study demonstrated that three to five years after transplant the corrected GFR was significantly higher in children who had received a paediatric graft, with grafts also doubling in size compared with no increase in size noted in adult grafts. Pape et al. therefore recommended that paediatric donor kidneys should be given to paediatric recipients.

This led to further studies being undertaken to confirm these findings. Goldsmith et al. [6] described how the scarcity of small paediatric donors required to size match donor and recipient would lead to unacceptably long waiting lists. They conducted a retrospective study between 1999 and 2008 of all paediatric renal transplants performed in their centre in Leeds, UK. Two groups, one comprising recipients of weight-matched and the other of mismatched donor grafts, were compared for one-year graft survival. They found no significant difference between the two groups, and concluded that adult sized kidneys with even a considerable donor-recipient weight mismatch can be safely transplanted into small paediatric recipients.

Dick et al. [34] retrospectively studied the impact of donor-recipient size mismatch on long term kidney graft survival in a large number of paediatric patients. They reviewed the United Network for Organ Sharing database from 1987 to 2010 and identified 1880 patients between the ages of 11 and 18 years who underwent renal transplantation. The patients were divided into two groups according to donor/recipient body surface area ratio (<0.9 and ≥0.9), and these were compared for graft survival rates. Analytical adjustments for potentially confounding factors such as cold and warm ischaemia times, ethnicity, gender, donor, and recipient ages were taken into account. Dick et al. found that body surface area ratio <0.9 conferred a statistically significant increased risk of graft loss and, therefore, proposed that appropriate size matching of donor-recipient kidneys confers better long term graft survival.

## 4. Conclusion

End stage renal failure in young children is a distressing condition, both for patient and family, and carries an important risk of morbidity and mortality. Transplantation is the mainstay of definitive treatment, but this is both logistically and technically challenging in the younger and smaller recipient.

A scheme to size match grafts and recipients would lead to unacceptably long waiting lists and is thus not feasible to meet the needs of our growing population. The alternative solution is to employ adult sized grafts; however, to perform this procedure, the surgeon may need to modify the technique of incision, approach, vessel anastomosis, and closure.

Postoperatively, the patient may experience life-threatening cardiovascular instability and graft hypoperfusion.

Undoubtedly, this procedure poses more challenges to the attending medical and surgical teams, but nonetheless, recent studies have shown a comparable outcome for children receiving both adult and paediatric kidney grafts, and many centres worldwide currently employ this practice.
